# Integrating media content analysis, reception analysis, and media effects studies

**DOI:** 10.3389/fnins.2023.1155750

**Published:** 2023-04-27

**Authors:** Ralf Schmälzle, Richard Huskey

**Affiliations:** ^1^Department of Communication, Michigan State University, East Lansing, MI, United States; ^2^Department of Communication, University of California, Davis, Davis, CA, United States; ^3^Cognitive Science Program, University of California, Davis, Davis, CA, United States; ^4^Center for Mind and Brain, University of California, Davis, Davis, CA, United States

**Keywords:** neuroscience, movies, media, naturalistic, media effects, content analysis, reception analysis

## Abstract

Every day, the world of media is at our fingertips, whether it is watching movies, listening to the radio, or browsing online media. On average, people spend over 8 h per day consuming messages from the mass media, amounting to a total lifetime dose of more than 20 years in which conceptual content stimulates our brains. Effects from this flood of information range from short-term attention bursts (e.g., by breaking news features or viral ‘memes’) to life-long memories (e.g., of one’s favorite childhood movie), and from micro-level impacts on an individual’s memory, attitudes, and behaviors to macro-level effects on nations or generations. The modern study of media’s influence on society dates back to the 1940s. This body of mass communication scholarship has largely asked, *“what is media’s effect on the individual?”* Around the time of the cognitive revolution, media psychologists began to ask, *“what cognitive processes are involved in media processing?”* More recently, neuroimaging researchers started using real-life media as stimuli to examine perception and cognition under more natural conditions. Such research asks: *“what can media tell us about brain function?”* With some exceptions, these bodies of scholarship often talk past each other. An integration offers new insights into the neurocognitive mechanisms through which media affect single individuals and entire audiences. However, this endeavor faces the same challenges as all interdisciplinary approaches: Researchers with different backgrounds have different levels of expertise, goals, and foci. For instance, neuroimaging researchers label media stimuli as “naturalistic” although they are in many ways rather artificial. Similarly, media experts are typically unfamiliar with the brain. Neither media creators nor neuroscientifically oriented researchers approach media effects from a social scientific perspective, which is the domain of yet another species. In this article, we provide an overview of approaches and traditions to studying media, and we review the emerging literature that aims to connect these streams. We introduce an organizing scheme that connects the causal paths from media content → brain responses → media effects and discuss network control theory as a promising framework to integrate media content, reception, and effects analyses.

## 1. Introduction

Media messages permeate our lives; they stimulate rich neurocognitive responses and serve important, much-debated functions within modern information societies. On average, we spend about 8 h per day consuming media (Twenge et al., 2019). Effects of exposure to media range from micro-level impacts on an individual’s memory, attitudes, and behaviors to macro-level effects on nations or generations (Bryant and Oliver, 2008; Larzabal et al., 2017). In short, we live in a world where media content flows through our brains much like blood through our veins.

In recent years, researchers have begun to use theories and methods from neuroscience to examine the neural mechanisms of media effects (Weber, 2013; Schmälzle and Grall, 2020a,b; Schmälzle, 2022). This approach is motivated by the fact that the brain is the biological organ underlying all media effects, regardless of whether the study is about movies, narratives (books and audiobooks), or other media types. After all, if a message did not arrive in a recipient’s brain, it could not have any effect. This notion of the brain as the central processor of media content is undisputed. It is what motivates the use of neuroimaging to study brain responses to media in the hope of revealing the actual mechanisms that underlie media’s effects on perception, attention, comprehension, affect - or whatever the focal topic of a concrete neuroscientific investigation that uses media may be.

However, while the promise of neuroimaging in this area is generally recognized, the complexity of the enterprise cannot be underestimated. Media are a highly complex kind of ‘stimulus’, actually, they are a sequence of a multitude of individual stimuli. Moreover, media evoke multiplex brain responses. And finally, media result in a mosaic of consequences - from short-term to long-term effects and from individual to collective outcomes.

**Figure 1:**
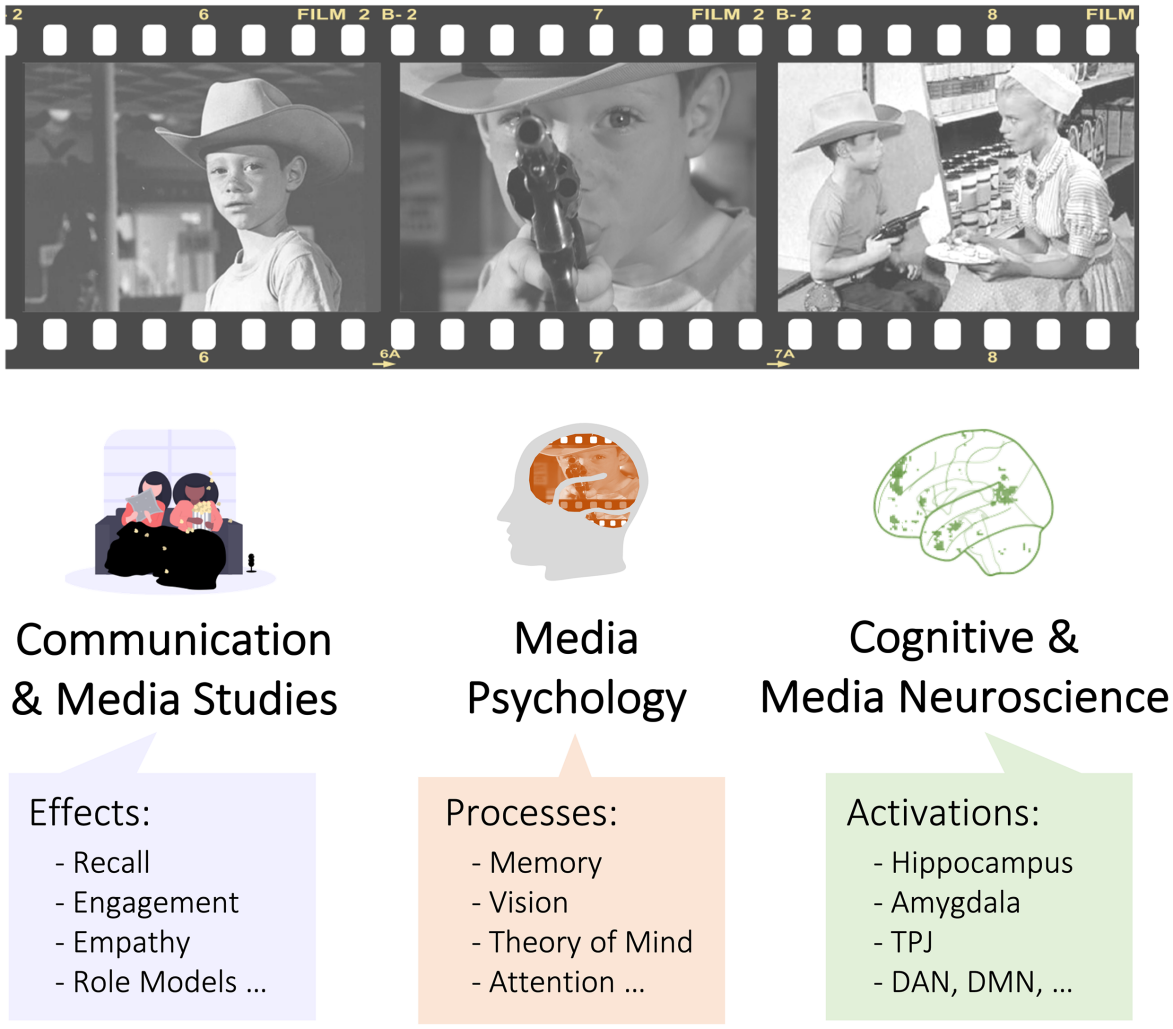
Connecting siloed fields of media effects, media processes, and media neuroscience. Traditionally, these topics have been studied by different academic disciplines.

Given this complexity, it is no surprise that multiple disciplines exist at the nexus of media and the brain. Researchers in the fields of communication and media studies have largely focused on issues related to media content and the effects of exposure to such content (Figure 1, left; Riff et al., 2014; Neuendorf, 2017). By comparison, psychology and media psychology investigate the cognitive processes that subserve media processing and effects (Figure 1, middle; Weber et al., 2008; Lang and Ewoldsen, 2010). By comparison, the cognitive sciences and cognitive neurosciences primarily use media as a tool for studying cognition and the brain (Figure 1, right; Spiers and Maguire, 2007; Hasson and Honey, 2012; Sonkusare et al., 2019; Vanderwal et al., 2019).

**Figure 2:**
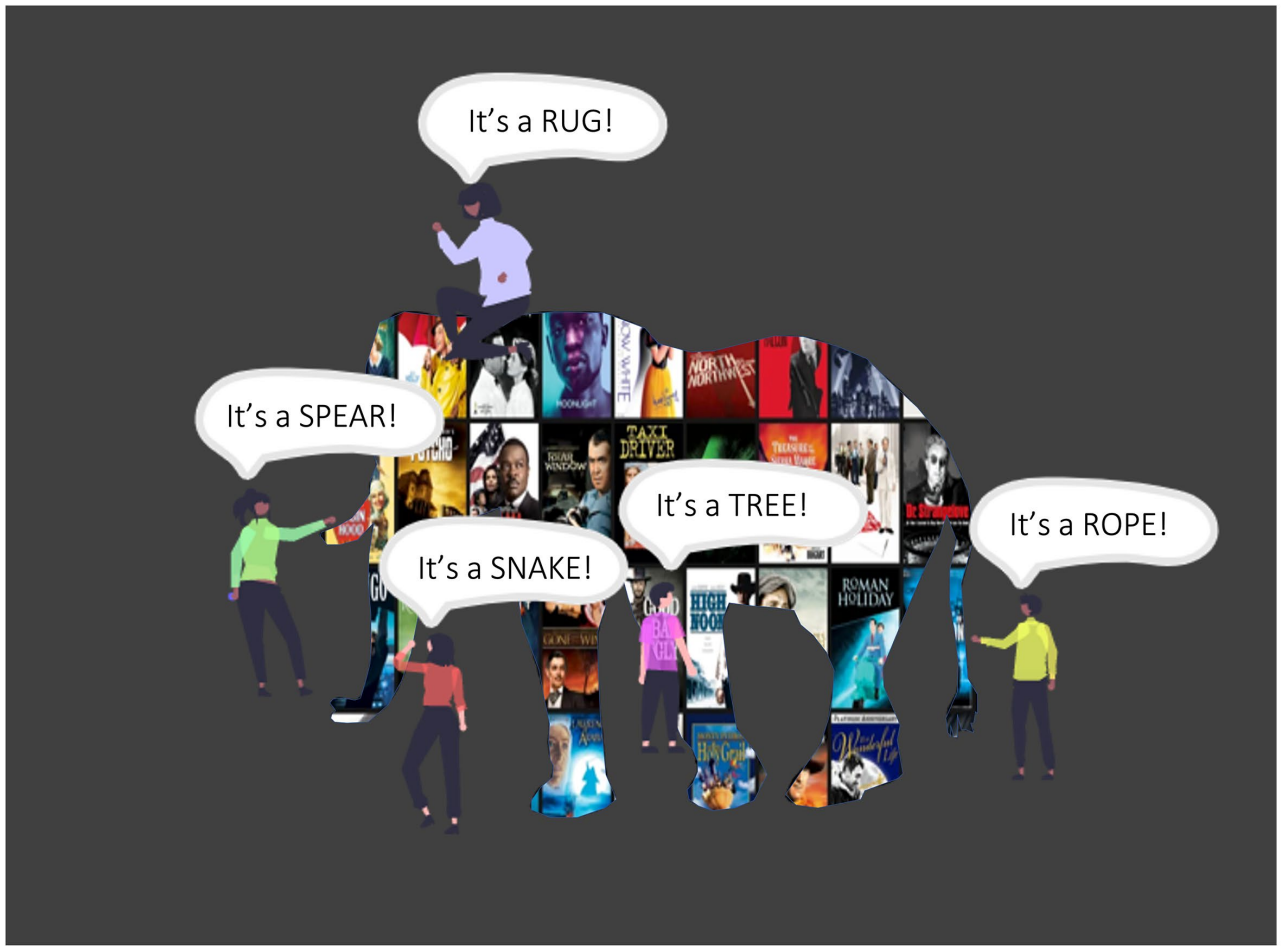
An elephant in a dark room. In this classic parable, people investigate an elephant in a dark room. Each can only feel part of the elephant and cannot identify the whole. Misunderstanding ensues.

Of course, these broad generalizations mask substantial disciplinary and topical heterogeneity such that inquiry surrounding media and the brain is a bit reminiscent of people feeling an elephant in a dark room (Figure 2): In this parable, each person brings their own experience and perspective to the endeavor of identifying the elephant, but each person is only able to feel just one small part of the large animal. In the same way, many different perspectives about media and the brain coexist - all valuable in and of themselves - but there is a lack of integration and a lot of confusion. In fact, early career researchers who consider working at the intersection of media and the brain will find themselves in a complex theoretical and methodological landscape that spans disciplines and even paradigms from the humanities, traditional STEM disciplines, and the social sciences. This state of affairs can make it difficult to see the proverbial elephant in the room, and one can almost ask oneself: If “naturalistic neuroimaging” or “movie fMRI” is the answer, what is the question (see Kosslyn, 1999)?

With this in mind, this article suggests a conceptual framework to integrate these disparate research streams of media effects, media processing, and media neuroscience. We begin by introducing and discussing each area and provide the logical division into *content analysis*, *reception analysis*, and *effects analysis* as an organizing scheme. Then, we suggest *network control theory (NCT)* as a framework with the potential for integrating these siloed traditions. We believe this framework can shed light on the elephant in the dark room and reveal causal mechanisms by which the content of media messages affects brain responses and how the resulting message effects in single individuals aggregate into media effects in large populations.

## 2. The arrow of causality: from media content to reception responses to media effects

**Figure 3:**
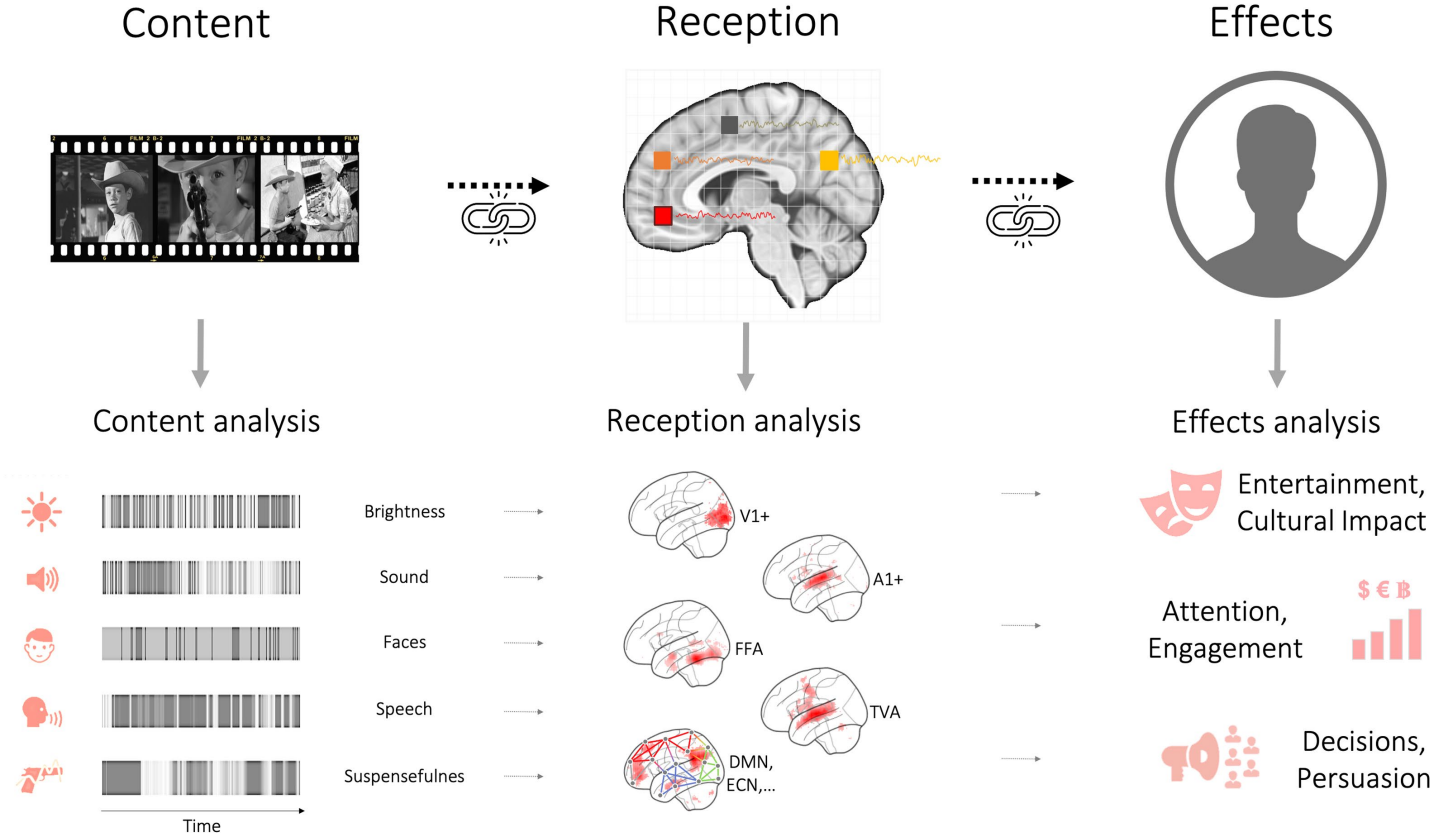
The arrow of causality from media content to reception responses to media effects. The bottom left panel illustrates how content analysis quantifies content (e.g., for use as a design matrix), allowing to map out brain systems responding to specific content elements. These can then be linked to effects of media consumption. Of note, reception responses to an incoming (causal) media stimulus can also be modulated by receiver state, background knowledge, beliefs, and so forth.

So far, we have discussed how different areas of disciplinary inquiry are largely organized around levels of analysis (media effects on individuals and society, media processing within individuals, neural responses within individuals). As this section will show, a framework organized around levels of analysis does not cleanly map onto a causal path that begins with exposure to media content and ends with media effects. In this section, we give an overview of our conceptual model that starts with media as a stimulus (a brief text message, an audiovisual movie, a social-media video clip, an audiobook) containing conceptual content that is analyzed by the brain and results in what has traditionally been called media or message effects (Figure 3).

### 2.1. Assaying the ingredients: media content analysis

Media are inherently content-rich and, therefore, complex. To demonstrate by selecting one possible example, consider movies. Among the most popular types of media, movies comprise multimodal content (images and soundtracks) that include a wealth of semantic and social-pragmatic dimensions that vary over time. The term movie emerged as a shortcut for moving images - essentially by stitching together photographs in rapid succession. For example, a typical Netflix HD movie streams about 3-7GB of data, containing over 100,000 individual frames, each containing many pixels. It becomes clear that if we consider the pixel-level information of any given movie, the information contained in a movie quickly reaches billions. These flickering pixels form the manifest content of the movie as it emerges from your TV screen.

Clearly, though, looking at movies as a multitude of pixels misses the point - just as it makes little sense to use a microscope to examine ink-saturated paper when reading a fiction book. Typically, when discussing movies, we mean their higher-order information, such as narrative and social-cognitive content. Clearly, we also do not remember the surface-level information (the pixels), but we recall and retell what happens to characters and the overall trajectory of a plot (like heroes and villains, or a rags-to-riches story, etc.; see Kintsch, 1998 for a similar argument about language comprehension).

**Figure 4:**
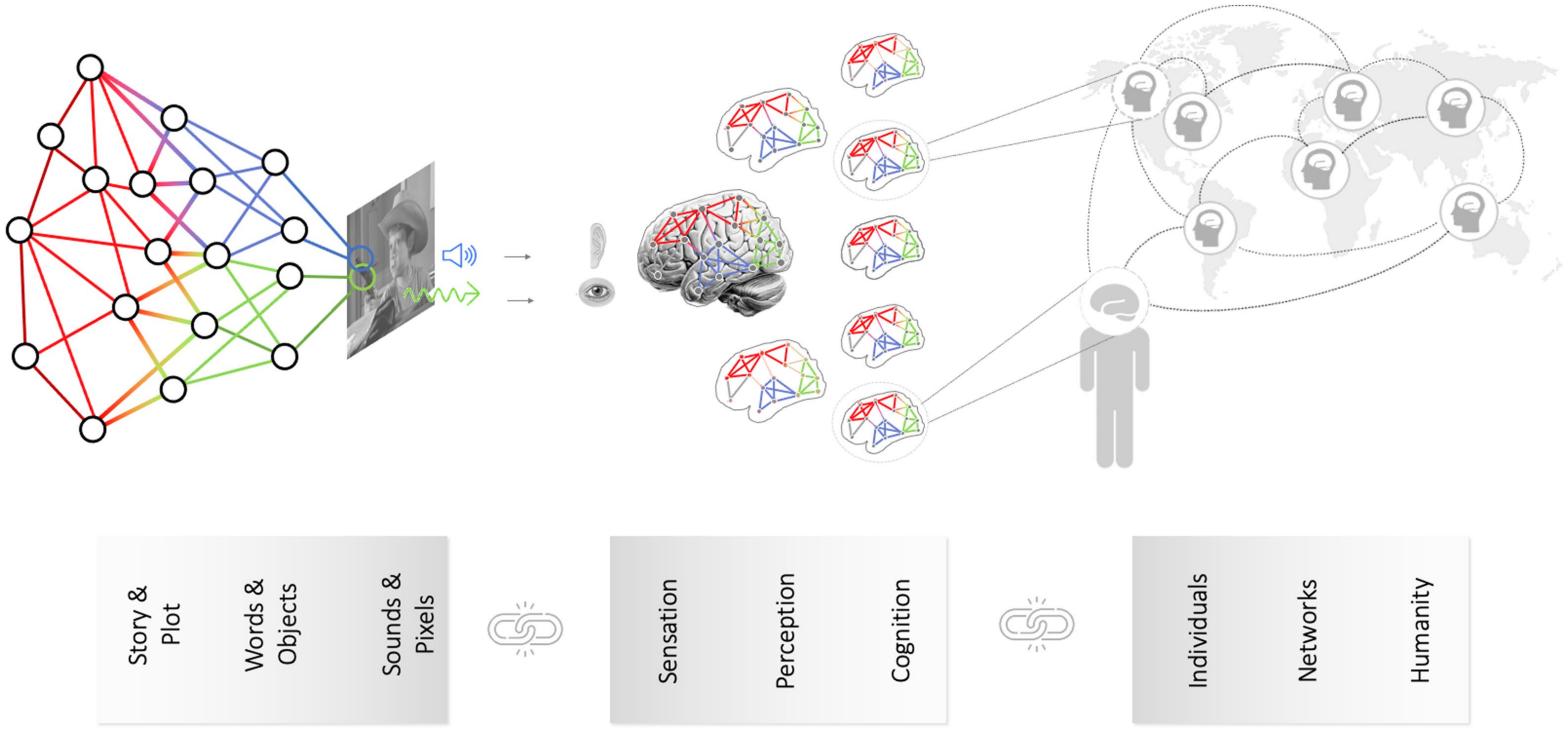
Linking hierarchies of content with matching reception mechanisms and integration with media effects. Content is a deceptively singular word, but it encompasses multiple layers - from manifest (e.g., pixels) to latent content (e.g., subtext, story morals). Understanding content as a network of information layers (left panel) allows for its comprehensive quantification, facilitating the identification of corresponding reception mechanisms. In a similar fashion, we can also integrate individual-specific neurocognitive processes during media reception to media effects and social levels.

Between the pixels as the lowest-level content features and the macro- or plot-level content features lie numerous intermediary-level features. For example, consider now the soundtrack of a movie (instead of the video track containing the pixels). At a lower level, a movie’s soundtrack is characterized by physical properties like its constituent amplitude, frequency content, etc. However, this all is embedded in a nested, hierarchical structure: Stretches of sound encode particular phonemes, which in turn represent words, words are nested in sentences, and a couple of sentences by one speaker are typically followed by a response from another speaker, reflecting a dialogue in a scene. The same case can be made for visual content (e.g., Hasson et al., 2008a). Thus, it becomes clear that *the content* of a movie - a deceptively simple singular word - actually encompasses multiple content elements that can be organized along a hierarchy of abstraction (see Figures 3, 4). Which specific content element is of interest to researchers often depends on their home discipline - just like in the elephant in the dark room parable. Arguably, since movies are largely created and consumed to entertain, the most relevant level is the plot level. Still, it is clear that all lower levels (sounds, words, sentences, paragraphs or pixels, images, scenes) are necessary to convey the plot-level content of a movie (or a book or whatever the media format).^1^

One way of quantifying all this higher-order and often latent content (or subtext) is through a procedure known as content analysis (Krippendorff, 2004; Riff et al., 2014; Neuendorf, 2017). Historically, content-analyzing movies and media more broadly was an arduous task. For example, the famous National Television Violence Study (Federman, 1995) relied on manual labor from human coders to annotate over 10,000 h of content over a three-year period. Most content analyses are smaller in scale, but the effort required is still a key bottleneck (Greenberg and Atkin, 1980; Masters et al., 1991; Hahn et al., 2017). Consequently, classical content analyses usually feature sparse sampling frames (e.g., the first 10 min of content from a sample of movies) that often incompletely describe the entire media corpus. Another, not unrelated problem, is that there is often substantial error in human annotations, which can be quite difficult or even impossible to resolve in some circumstances (Weber et al., 2018).

Advances in computational analysis make this task much faster, scalable, feasible, and accurate. The catch, however, is that computational analyses are currently only able to quantify manifest lower- and mid-level features of the content-abstraction hierarchy. We assume that readers will be familiar with the explosion of research on natural language processing and computer vision. As of 2023, computers can automatically quantify many sound characteristics (such as energy and pitch) and even transcribe spoken content into writing (Radford et al., 2022), and in the visual domain, they excel at quantifying image properties, recognizing objects, or even detecting actions in image sequences (Rohrbach et al., 2017).

These advances can be leveraged to analyze media content in a rigorously quantitative fashion and at scale. For instance, researchers have used face-detection systems to detect characters in movies or natural language processing (NLP) techniques to identify characters from scripts, both of which can be used to create character networks (e.g., communities of characters that co-occur in the same scenes; Hopp et al., 2020; Baldwin and Schmälzle, 2022; Malik et al., 2022) or to create time-locked explanatory variables for neuroimaging analyses. Or researchers have used NLP techniques to study moral language in media (Weber et al., 2018). Perhaps the most systematic yet still young approach in this area is the NeuroScout platform and the related pliers python package (McNamara et al., 2017; De la Vega et al., 2022). NeuroScout provides easy access to machine-learning methods capable of automatically extracting hundreds of features that range from the very concrete (like root mean squared amplitude of the sound signal) to more abstract ones (like concept-level image tags from Google’s or Clarif.ai’s computer vision systems).

Overall, computational tools for extracting content features are immensely promising for studying content in a rigorously quantitative and automatic manner. However, we must acknowledge that even the most advanced machine-learning systems fail to achieve human-level understanding (Marcus and Davis, 2019; McClelland et al., 2020). In fact, even though impressive progress is made in modeling so-called common sense knowledge, current systems still fall short in many regards when it comes to coding abstract categories of content, such as sarcasm or humor, or detecting sequential narrative information related to story schemata or scripts, or visual action depictions (Vicol et al., 2018; Choi et al., 2021; Zellers et al., 2021). Taken together, automatic approaches can excel at quantifying lower- and mid-level properties of content, but they still face a barrier (Karpathy, 2012; Mitchell, 2020) when it comes to analyzing higher-order media content.

Said differently, the content of a movie (or other media) can be analyzed very concretely and efficiently in terms of physical properties, such as brightness and contrast, and also for intermediate levels, like the presence of objects, such as guns and faces. At a yet more abstract level, however, the movie has an event structure (separated by cuts) and a plot that conveys the overall narrative. This type of abstract content is currently much harder to quantify, even with advanced machine learning and NLP techniques. Indeed, we often find ourselves resorting to psychological terms to describe content-level properties whose “ingredients” in content remain somewhat unclear, such as the ‘suspensefulnes’ of a movie to describe its potential to elicit suspense (see Cummins, 2000). However, it is clear that these content elements matter for a movie’s impact on viewers’ brain responses.^2^

The upshot of all this is that if our goal is to understand the effects of content of the brain, then a diverse and rapidly improving toolkit for quantifying media content already exists for more concrete features, and we can rely on traditional human content annotations to quantify higher-level aspects of content that are still beyond the capabilities of computational tools. In the next section, we discuss how this quantified content is the key to deciphering the brain responses.^3^

### 2.2. Reception analysis: how brains respond to media

It is clear that media content’s arrival in the brain sets forth a cascade of reactions (Kepplinger, 1989; Bryant and Zillmann, 1990; Potter and Bolls, 2012; Schmälzle and Grall, 2020a). Just like we started our analysis of movie content at the pixel level, we can begin our quest into the brain at what can be considered the neural counterpart of the pixel: an individual cell (rod/cone) in the receiver’s retina that gets stimulated by light and converts the televised movie’s signal into a neural impulse. Due to space limits, we cannot trace this signal’s neural itinerary in fine detail, but a rough sketch goes like this: From the retina, information travels along the optic nerve into the thalamus, gets relayed in the lateral geniculate nucleus, and arrives *via* the optic radiation in the primary visual cortex (Mesulam, 1998; Chalupa and Werner, 2003; Fuster, 2003; Poeppel et al., 2020), and so forth. The seminal work by Hubel & Wiesel on receptive fields provides perhaps the most concrete examination of content-extractors (or feature detectors) in the brain; that is, neurocognitive mechanisms that match certain content elements, like oriented lines, edges, or motion (Hubel and Wiesel, 1962).

However, just like with the analogy of trying to read a book with a microscope, studying movies as purely visual stimuli that activate the retina and V1-edge-detectors runs the risk of missing the point: We clearly do not watch movies simply to obtain visual stimulation, and we do not read or listen to books solely because we like letters and sounds, or processing any of the intermediary representations like objects, action sequences, or speech. Instead, we typically use media to engage their higher-level, albeit more difficult to quantify, content.

Few researchers would question the statement that “content is key” for understanding how media impact the brain. However, looking into the emerging literature on media and neuroscience, it is apparent that content is often simply ignored. In some ways, this is understandable. Modern neuroscience already requires extensive training in neuroanatomy, physiology, physics, statistics, engineering and signal processing, psychology, philosophy, programming, high-performance computing, and so on, such that there is little time left to also train in scholarship on complicated and sometimes even poorly specified content features that come with media stimuli (e.g., narratives, characters). Similarly, when using media as stimuli, it is not always so clear exactly what needs to be accounted for in either experimental design or statistical analysis. Should we account for luminance? Sound amplitude? The presence of faces? If so, how? The difficult answers and unappealing tradeoffs associated with these questions have spurred clever solutions optimized for designing around all of this complexity. Such approaches include calculating intersubject correlations (ISC; Hasson et al., 2004, 2008b), or borrowing other methods from resting-state fMRI, dynamic causal analyses (e.g., Granger causality or DCM methods), or introducing other advanced tools to decipher entangled brain responses (Di and Biswal, 2020; Van Der Meer et al., 2020; Busch et al., 2022).

It is not our goal to criticize this research as it has already led to important new discoveries about the brain. Nevertheless, these approaches are largely content-blind. We argue that without an equal appreciation of the content, this endeavor will yield only limited insights (see Okdie et al., 2014, for a parallel argument about media psychology). After all, it is clearly the content where the causal arrow originates that evokes the brain responses. Thus one should devote equal sophistication to content analysis as to reception analysis (i.e., analysis of neural or other types of data).

Not all neuroimaging analyses are content-blind, though. In fact, some go to great lengths to quantify or manipulate content. However, we claim that even these approaches are still limited when it comes to identifying the kinds of higher-level content elements that prompt conceptual and affective reactions to media and drive media selection and consumption behavior. For example, in studies of natural vision, movies are increasingly adopted as stimuli because they depict relatively natural scenes (except for things like cuts and blends; Hasson et al., 2008c; Çukur et al., 2013). Such studies also tend to do a great job quantifying aspects of content that are relevant to their area of study, like meticulously annotating visual content properties such as contrast, individual objects, and so forth, or manipulating content *via* scrambling (Hasson et al., 2008c; Çukur et al., 2013; Huth et al., 2016b; Wen et al., 2017). Studies like these make great use of movies as an experimental stimulus, and they can serve as role models for how content analysis can inform reception analysis. These studies represent the kind of work that examines carefully one specific part of the proverbial elephant (e.g., visual processing). As such, they are extremely valuable for understanding vision. However, although vision clearly is central to movie viewing and the entertainment experiences it produces, vision alone is only one piece of a larger mosaic of movie-evoked brain responses. Moreover, to the extent that higher-level content properties (such as suspense fluctuations in a movie) impact attention, it is probably the case that the measurements might be biased (e.g., Van Berkum et al., 2009; Gantman and Van Bavel, 2014; Schmälzle and Grall, 2020a,b).

Much like the visual neurosciences have begun to adopt media as a more naturalistic alternative to traditional stimuli, neurolinguistics has also begun to embrace media (like stories, audiobooks, and movies with dialogue). In the early days of neuroimaging, language studies were notoriously artificial single-word studies (e.g., using sparse sampling event-related designs). The trend towards more naturalistic neuroimaging prompted an upsurge of studies using natural, running speech as stimuli - often taken from audiobooks and similar story-based media formats. Like their counterparts in the visual domain, neurolinguistics studies do a great job at annotating word-level linguistic properties, such as word length, frequency, syntactic role, or even basic semantic aspects (e.g., GloVe or Word2Vec embeddings) and relating these to the stimulus-evoked brain activity in a forward-inference manner (Lerner et al., 2011; Huth et al., 2016a; Broderick et al., 2018). As this trend advanced, the stimulus characteristics that were coded became more nuanced; for instance, it has been demonstrated that decoding results become better if one uses sentence-based embeddings as opposed to word-level-only embeddings. However, the key point is that these neurolinguistic studies also struggle to consider content elements that go beyond the linguistic level (McClelland et al., 2020; Arana et al., 2023). However, just like reading a book with a microscope, we claim that we do not consume stories because they provide linguistic stimulation. Rather, it is the supralinguistic content and the responses this evokes that are critical: stories entertain, satisfy social needs, pique our curiosity, and so forth.^4^

A still small but growing number of studies attempt to link higher-level media content, which influences post-perceptual processes like attention, semantic comprehension, and particularly affective and social-cognitive responses, to brain responses (Hasson et al., 2008b; Yeshurun et al., 2017; Richardson et al., 2018; Tikka et al., 2018; Nguyen et al., 2019; Schmälzle and Grall, 2020a,b; Baek and Parkinson, 2022; Grady et al., 2022). For example, it is well known that movies are highly social in content and that their social and affective content is key to why we engage with them in the first place. In fact, movies are bursting with depictions of social interactions, including love, aggression, betrayal, etc. - and viewers take an intense interest in the fate of characters (Bryant and Zillmann, 1990; Oatley, 2002; Tannenbaum, 2014). Because of this, movies and other fiction-based media are almost ideal tools for studying social cognitive processes like empathy, perspective-taking, trait inferences, and so on (Vorderer, 1996; Klimmt et al., 2006). These characteristics of movies are increasingly recognized by neuroimagers interested in the neural basis of such processes (Salmi et al., 2013; Byrge et al., 2015; Richardson et al., 2018; Nguyen et al., 2019; Broom et al., 2021; Chang et al., 2021), even beyond human neuroimaging (Mantini et al., 2012; Sliwa and Freiwald, 2017).

Similarly, these social-cognitive responses to movies are intimately interwoven with affective reactions. For instance, viewer affect reliably tracks character victories and failures, good fortune and suffering, trials and tribulations such that audiences experience strong participatory responses (e.g., goosebumps during the hero’s victory at the end, crying during ‘all is lost’ moments when it seems that the hero is doomed to failure). In fact, it has been said that Hollywood is - at its core - a giant experimental psychology lab specializing in creating emotional stimuli that can effectively affect mass audiences. Likewise, Alfred Hitchcock, the famous master of suspense, described his profession as “based on an exact science of audience reactions” (Hasson et al., 2008a). Because of this capacity, entire genres of movies are devoted to catering to certain segments of the affect spectrum, and a few neuroimaging studies have explored such phenomena. For instance, suspense movies take audiences on an emotional rollercoaster that blends future-oriented cognitions like hope and anxiety (Bezdek et al., 2017; Schmälzle and Grall, 2020b). Action movies can stimulate intense bursts of arousal (Hermans et al., 2011; Kautonen et al., 2018). Comedy tickles our funny bone (Sawahata et al., 2013; Amir et al., 2015; Jääskeläinen et al., 2016; Schmälzle et al., 2022), drama/tragedy deals with human responses to suffering (Raz et al., 2014, 2016). And, while often hushed up, pornography is certainly quite powerful in stimulating experiences (Prause et al., 2015; Schmälzle et al., 2017; Chen et al., 2020; Grubbs and Kraus, 2021).

In sum, it is clear that media feature a host of content that can elicit and precisely steer social-cognitive and affective processes. In fact, due to this capacity, media are very promising to study the neural basis of these phenomena in a way that is more appropriate to their nature than, say, event-related studies of single words, affective images, and so forth (Hasson and Honey, 2012; Saarimäki, 2021).

The challenge, then, is to quantify the social and affective content characteristics to be able to unlock its mechanism of action *via* neuroimaging. The studies presented above are in an advantageous position because the content properties that we care about are relatively well understood and can be coded straightforwardly (as done in the NeuroScout system or *via* the Matlab vision toolbox or some natural language processing toolbox). By contrast, when the research focus is on social-cognitive and affective phenomena, the task of coding the conceptual content is considerably more difficult,^5^ although some clever ways exist to attempt to parametrize these more challenging factors (Heider and Simmel, 1944; Meyer et al., 2019; Nguyen et al., 2019). But it is clear that if we ignore higher-level content altogether, then we cannot expect to meaningfully relate brain responses to their elicitors - at least not beyond relatively simple sensory-perceptual brain responses, and if top-down attention comes into play, even these will get affected. This is the problem with ‘content-blind’ neuroimaging.

### 2.3. Media effects: how media influence individuals and large-scale populations

The last link in the causal chain from content to reception is the question of how exposure to media changes memories, attitudes, or behaviors. The term media effects refers to these psychological or behavioral outcomes of stimulation with media. Of note, the term media effects is used to refer to individual-level as well as population effects (Bryant and Oliver, 2008). The latter clearly depend on the former, but in practice, they tend to be studied by different research communities who focus either on micro- (intraindividual) or macro (social) levels of analysis.

The origin of the field can be traced back to social scientific research in the 1920s and 30s, which is the era when the first distant mass media (radio, TV) emerged. Historically, the field has swung back and forth between periods in which researchers postulated relatively strong media effects and those of weaker effects. For example, in the period between 1920 and 1950, much research attention centered on the putatively strong influence of propaganda (Hovland and Lumsdaine, 2017). Modern efforts showcase that media effects tend to be smaller in nature and more contextually dependent (Lang, 2013; Rains et al., 2018). Nevertheless, and despite substantial evidence to the contrary, today’s pressing topics like radicalization, fake news, deep fakes, and the influence of social media are often cast in overly simplistic terms and assume overly powerful effects. Neuroimagers looking to use media as stimuli should recognize that, contrary to common perceptions, media effects tend to be quite small in practice.

The list of media effects and media effects theories is too long to discuss here. Still, a partial list of interesting phenomena and theories includes, e.g., the third-person effect - the belief that media influence others more than oneself (Perloff, 2002). Readers are likely familiar with the famous Bobo Doll Study that helped give rise to Social-Cognitive or Social Learning Theory (Bandura, 1977). Central to this theory is the notion of observational learning and role models - both of which can occur during media consumption - and therefore Social Cognitive Theory is widely used to explain social media effects (Bandura, 1994). Similarly, Affective Disposition Theory (Zillmann and Cantor, 1972; Raney, 2004) links characters and plot elements to affective audience responses. There are, of course, many other interesting effects and theories of media influence to highlight, but for the sake of space, we refer readers to key reference works (Zillmann and Vorderer, 2000; Bryant and Oliver, 2008; Littlejohn and Foss, 2009; Nabi and Oliver, 2009; Dill, 2013; DeFleur, 2016).

In essence, any result of media stimulation could be considered as a media effect, whether it is short-term memory (e.g., recalling last night’s news), long-term memory (e.g., remembering a childhood TV show), a change in attitude, a belief (e.g., being more open to immigration after watching a refugee drama), or behavior (e.g., donating money to charity after viewing an ad). These effects are often linked to their elicitors in content, but how the brain mediates between content and effects has traditionally been ignored. Instead, because neuroimaging measures were unavailable until recently, researchers had to rely on self-report methods that were usually taken after the media consumption ended (Lang, 2014).

Critically, media effects are not only studied in single individuals but often with an eye toward aggregate audiences. The field most closely associated with this perspective is mass communication. In brief, mass communication describes a *one-to-many* mode of communication in which the same message is sent out to multiple recipients. For instance, early mass media were newspapers where the same article would be read by all readers. Radio marked another milestone, then most notably followed by Television. And, although social media has now upended the traditional “one-to-many” model of mass communication, providing a many-to-many mode of communication instead, it is still true that a single social media message can be sent out to a large audience, and the brains of audience members would then still respond to the same message (Schmälzle and Grall, 2020a,b; Gong et al., 2022).

Given the important effects media can have on the masses and public opinion (Lippmann, 1922; Noelle-Neumann, 1991), it is clearly of interest to examine how reception responses relate to such large-scale media effects. In other words, might media-evoked brain responses allow researchers to predict subsequent effects? Indeed, several emerging neuroimaging studies (and a large body of non-neuroimaging studies from the social sciences more broadly) have begun to examine this question. For instance, Hasson et al. showed that brain imaging data captured during viewing could predict memory, a very concrete and clear-cut media effect (Hasson et al., 2008a). Falk et al. showed that brain responses to health messages could predict message-consistent behavior change at later points (Falk et al., 2010), and several other articles examine effects related to persuasion, broadly defined, or engagement with and sharing of messages in social networks (Weber et al., 2015a; Baek et al., 2017; Huskey et al., 2017; Coronel et al., 2021). These studies point to the potential of using brain imaging data to predict individual-level outcomes, that is, how to link reception responses captured in individuals to the ensuing media effects.

Another intriguing twist for using brain imaging data is to predict collective outcomes. By that, we mean that it is possible to record the brain’s responses during reception from a smaller test audience and link them to aggregate outcomes in larger groups (Berkman and Falk, 2013). For example, in the neuroeconomics literature, researchers have predicted the cultural popularity of music from brain responses (Berns and Moore, 2012). Similarly, Dmochowski et al. (2012), used brain responses to SuperBowl commercials to predict online engagement (tweet volume; Dmochowski et al., 2014), and Falk et al. used brain responses to health messages to predict campaign success (call volume to an anti-smoking quitline; Falk et al., 2012).

The broader reasoning behind these efforts, which connect the brain responses of single individuals or small groups to large-scale population-level media effects, is based on the one-to-many mass communication logic: A message is sent out and processed by multiple individuals comprising an audience. If a given test audience is representative of a larger population, their brain responses can serve as a potential predictor of aggregate outcomes. That this works is just as logical as it is logical to use self-reports from samples to forecast larger outcomes (Knutson and Genevsky, 2018). At present, this approach has been used only in a few studies. Still, given the desirability of movies and media as stimuli, we can expect that many others will follow: After all, movies often even galvanize culturally shared, long-lasting collective memories (e.g., the famous shower scene in Hitchcock’s Psycho), suggesting that these effects have a shared basis in the brains of people who saw the specific footage (see, e.g., Kauttonen et al., 2018 for a neuroimaging study of key-frames). The same logic can also be applied to study how movie content produces any kind of convergent audience response, from collective suspense and fear during a horror movie to collective laughter during comedy (Schmälzle, 2022; Schmälzle et al., 2022).

Taken together, media effects are clearly consequential, of enormous interest to social scientists, and one of the most attractive areas that neuroscience researchers would like to seize. Especially the widespread ability of digital data (e.g., time-locked comments during movies and shows, social network metrics; Dmochowski et al., 2014; O’Donnell and Falk, 2015; Ni and Coupé, 2023) increases, there are unprecedented opportunities to link neural data to media effects. However, doing so in a meaningful way will - again - require keeping an eye on the content that starts the logical sequence from media content to brain responses to media effects. Said differently, we can only hope to explain media effects if we trace them back to the preceding brain responses and these, in turn to their elicitors in content.

To summarize, the previous section presented content analysis (2.1), reception analysis (2.2), and effects studies (2.3), arguing that these domains stand in a logical relationship with each other. And in each of these sections, we have pointed to the ways researchers have typically engaged in linking media, neural responses, and effects. These projects, while groundbreaking in their own right, often only investigate a subset of the causal chain from media content to reception responses to media effects. In what follows, we introduce Network Control Theory (NCT, Liu et al., 2011) as an integrative analytical framework that is well-suited to help further integrate these domains.

## 3. Network control theory: examining how media bring brains into specific states

In this article (and the special issue in which it appears), the brain takes center stage as the organ of media reception; that is, the site of action where complex content sets forth the activities that ultimately produce media effects. However, it is clear then that quantifying content is only half the battle - the other half deciphering the brain’s reactions to it. This, in turn, requires a general theory of brain function to motivate an analytical framework for studying content-brain relationships. Our model of brain function is based on current cognitive neuroscience research that views the brain as a complex, hierarchical network (Mesulam, 1998; Fuster, 2003).^6^ Entry-points into the network and its lower-level nodes (the eye, retina, optic nerve, LGN, and V1+; or the ear, cochlea, auditory nerve, olivary colliculi, and A1+) are relatively localized, and they correspond rather directly to specific lower-level content features (e.g., Hubel & Wiesel-type feature detectors). Subsequent layers of neural processing, however, tend to be more distributed, which calls for more multivariate analysis methods.

Over the past decade, network-based multivariate methods have been applied to neuroimaging data, and several large-scale brain networks have been identified (e.g., Medaglia et al., 2015). However, much of this work has been based on data captured in the so-called resting state, i.e., with participants only lying in the scanner. While this work has led to substantial and important insights, it is clear that the unconstrained nature of the resting state task is a limiting factor. By contrast, movies and media more broadly are ideal candidates to advance this research: They provide a rich and relevant stimulus for participants and one that is controlled insofar as it provides exactly the same input for everyone. Moreover, media can steer neurocognitive responses related to perception, attention, memory, and emotion, and it is this property that makes them ideally suited for studying cognitive neuroscience but also relevant for social science research trying to understand their mechanisms of influence. With this in mind, we will next introduce a mathematical framework - Network Control Theory - that uses external control forces (here: a movie and its content) to steer networked systems (here: the brains of audiences exposed to the movie).

### 3.1. What is network control theory?

**Figure 5:**
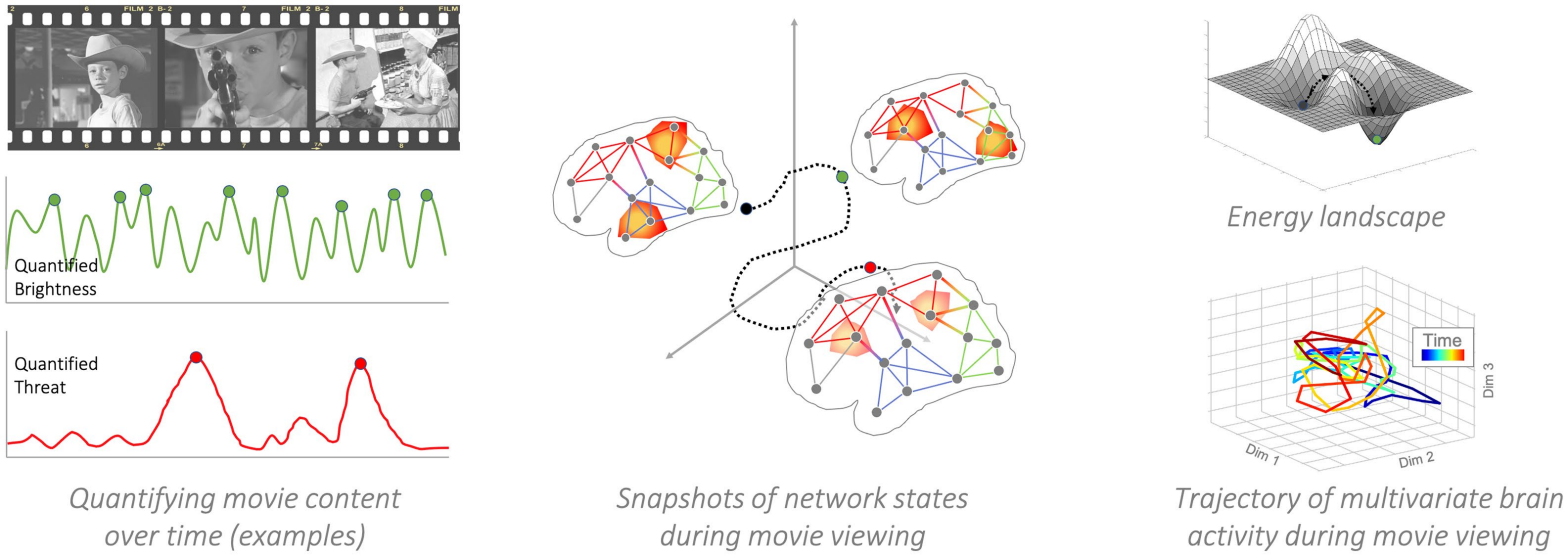
Network Control Theory. Left panel: Movie events are the causal forces that push the brain (or brains of entire audiences) into different states. For instance, the sudden reveal of a betrayal will likely engage theory-of-mind processes associated with social-cognitive brain networks. The depiction of a potential shooter approaching an innocent victim will engage affective systems. In this particular example, brightness and threat could be used as a time varying energetic source to use in a control theoretic analysis. The idea being, that each is analogous to an energetic source that should impact specific nodes (visual cortex, PFC, respectively) differently, and have different cascading impacts on time-varying network topology. Middle panel: A snapshot of network states in a single individual. As the individual views the movie, their brain responds to the time-varying content. Visual changes will prompt visual networks to come online and reconfigure (the example focuses only on brightness, but other visual features could be modeled, such as the presence or number of faces, contrast, objects, etc.). In parallel, higher-level content elements (though conveyed *via* the concrete sensory-perceptual features) prompt changes in networks related to executive control, salience processing, etc. Right panel, top: Example of an energy landscape in which valleys represent equilibrium states. By application of control energy, the brain can be pushed to leave one state and settle down in another. Note that the actual landscape has a higher dimensionality. Right panel, bottom: Example of a multivariate brain activity trajectory from actual movie-viewers. The input movie was Bang-Bang! You’re dead by Alfred Hitchcock. Brain activity from different regions (268-dimensional) is embedded in a lower-dimensional (3-dimensions) space (Heusser et al., 2018). Color represents time. As can be seen, the time-varying movie content steers brain activity into different positions.

Network control theory is a branch of control theory in engineering and a subfield of the larger network sciences (Gu et al., 2015). It deals specifically with the question of how networked systems can be controlled. What does it mean to *control* a network? Simply put, network control theory is a computational model that specifies if and how interventions, and their corresponding energetic costs, drive complex systems between different topological organizations with different energetic requirements (Muldoon et al., 2016; Tang and Bassett, 2018; Kim and Bassett, 2020; Lydon-Staley et al., 2020). More specifically, a given network topology requires energy costs to maintain.^7^ Networks can shift between different topological organizations, each with a different energetic requirement and these topological shifts can have their own energetic requirements, as well (see Figure 5).

To make this idea concrete via example, imagine a system of highways connecting several cities. The topological organization of this series of highways requires energy to construct and requires energy to maintain. Imagine now that the topology is updated; a new highway is built and added to the existing network. Constructing the new highway will also require energy, as will maintaining that new highway. Introducing the new highway might also alter the energetic maintenance costs of the highways that already existed in the network (e.g., the new highway may increase or decrease traffic flows on preexisting highways). Similar ideas can be applied to the brain as a network, although empirical evidence about energetic costs and network structure is less robust. For instance, the creation and maintenance of specific connections (white matter pathways) in the brain’s network are associated with energetic costs, and the topological structure is associated with the kinds of states and functions that the network can settle into and perform (e.g., Margulies et al., 2016).

Network control theory can be used to calculate a number of metrics that describe these energetic costs. Importantly, some network topologies are easier to reach - requiring less energy to obtain - than others. How can these energetic requirements be quantified? One of the most common approaches is known as *controllability.* Controllability is the ability to transition a network from one topological organization to another *via* external energetic input (Kalman, 1962). This controllability metric can be further subdivided into *average controllability*, *modal controllability*, and *boundary controllability*.

*Average controllability* (Shaker and Tahavori, 2013) describes how much energy needs to be applied to the system to transition the system into a different topological organization. Higher average controllability means that less energy input is necessary to drive the system to different topological organizations. One constraint on average controllability is that it only captures how much energy it takes to move the system into easily reached topological organizations. *Modal controllability* (Hamdan and Nayfeh, 1989) accounts for circumstances where it takes substantial energy to transition the system into a hard-to-reach topological organization. Finally, *boundary controllability* (Bassett et al., 2013) identifies nodes within a network that, when targeted with energy, can elicit connection or disconnection among other nodes in the network. Together, these metrics provide insight into the energetic costs and target nodes necessary to drive a network from one topological organization to another.

### 3.2. How has network control theory already been applied?

Network control theory has been increasingly applied to study the controllability of structural and functional brain networks (Medaglia et al., 2017), but it is not confined to brains alone. Instead, it is also perfectly feasible to apply network control theory to social or psychological networks (Abelson, 1964; Cremonini and Casamassima, 2017; Borsboom et al., 2021). For example, in neurology and neuropsychology, one can use network control theory to examine how strokes at specific anatomical (structural) sites affect cognitive (functional) processes (Popova et al., 2022). Similarly, in the case of social networks, it becomes possible to ask how structural changes affect function (Proskurnikov and Tempo, 2017, 2018). For instance, how do changes in leadership structure impact a group, its communication, and ultimately performance? Finally, turning to psychological networks such as attitude and belief networks, network control theory enables simulating how targeted influence (e.g., message-based persuasion attempts geared towards a specific belief) would impact the targeted belief, its associates, and the belief network as a whole (Schlicht-Schmälzle et al., 2018; Chambon et al., 2022).

Turning specifically to brain organization, network control theory has revealed some crucial findings about brain structure and function. Possibly most important is that the brain’s intrinsic architecture, that is, the white matter fiber tracts connecting gray matter structures, facilitate controllability in different ways. In a pathbreaking study, Gu et al. (2015) demonstrated that different neural subnetworks had different levels of controllability. For instance, the default mode network has a topological organization that facilitates transitions into other easily reached topological organizations. By comparison, other subnetworks (e.g., fronto-parietal control networks) are better suited to facilitate transitions into difficult-to-reach topological organizations. These controllability characteristics appear to guide high-level cognitive and behavioral responses within organisms (Rouse et al., 2021).

### 3.3. How can network control theory integrate media content with reception responses and media effects?

How can network control theory be applied to the media content → brain reception mechanism → media effects framework presented above, and what can we gain from it? In a nutshell, our core argument is that under a normal mass communication regime (i.e., one-to-many: same message, many recipients), the arrow of causality starts with the message content. Therefore, understanding the content is the key to understanding downstream effects.^8^

To give an example, consider the case of a movie that contains a morally evocative event, such as an innocent person being shot and killed.^9^ Such key moments of the story (Wilensky, 1983) evoke predictable audience reactions that are highly consistent across viewers (Hasson et al., 2008a; Dmochowski et al., 2012; Naci et al., 2014; Schmälzle and Grall, 2020a,b). It is clear that flickering pixels, moving images, and so forth are required to transmit the movie into peoples’ brains. However, the main “effective ingredient” of this content sits at a higher level of plot abstraction. We also know that filmmakers, screenwriters, and fiction authors are very skilled at “pushing” people into certain psychological states (see Figure 5). In fact, even the designation ‘director’ clearly alludes to the potential to exert control, that is, by influencing the content creation process in such a way that certain audience reactions follow predictably.

With neuroimaging, we can now capture how brain networks reconfigure dynamically during movie watching, such as how movie events trigger attentional reorienting responses, how close-up shots of protagonists are important events that evoke theory-of-mind processing, or how morality violations engage brain networks involved in emotion and socio-moral cognition. If we can successfully integrate these higher-level layers of the media’s content with the more easily quantifiable characteristics of content that engage sensory and perceptual brain systems, then we can hope to close the explanatory gaps between movie content, reception response, and media effects under one cohesive framework.

To make this all more concrete, consider the following example: We know that simple narratives are easier to follow than complex ones. From a cognitive perspective, we further know that following a complex narrative taxes working memory. Neurally, we know that working memory is associated with (although not in a 1:1 fashion) activity in the executive control and default mode networks. Thus, at a very simple level, we might examine network controllability metrics for different narratives that vary in complexity, and we could expect that simple vs. complex narratives are associated with different controllability values.^10^ Further, we might also ask if these controllability values can be used to predict box office revenues of a given narrative, much in the same way as Dmochowski and colleagues (Dmochowski et al., 2014) used neural reliabilities to predict audience preferences. In this case, we would link a high-level media content characteristic (plot complexity), with an equally high-level reception response (controllability), and media effect (box office revenue, a measure of popularity).

Of course, it should also be possible - and maybe more interesting - to apply the approach to a single movie to examine finer-grained elements along the media content, reception response, and media effects pathway. In this case, the time-varying properties of the movie would comprise the input to the system, i.e., the energy that is applied to the network. Mathematically, this can be modeled *via* impulse response models (Blaauw et al., 2017) when targeting a single node or more generalized control models (Tang and Bassett, 2018) that target multiple nodes in a network (for a review, see Lydon-Staley et al., 2020).

The question, then, is what type of media content we should model, to what node or nodes (targets) in the network the resulting energy would get applied, and what sort of outcomes we might expect? Although answers to these questions remain speculative because - to our knowledge - NCT has yet to be applied to content-rich media (as opposed to simpler stimuli and tasks), the cumulative body of knowledge from sensory and cognitive neuroscience, combined with nearly six decades of entertainment research and mass communication research can offer direction.

Starting with basic sensory and perceptual features, we can extract these in much the same way as is currently done for topical studies of vision, audition, or language (e.g., Kauttonen et al., 2015; McNamara et al., 2017), and we can relate quantified content properties (e.g., over-time variations in brightness, sound energy, etc.) to brain imaging measures. To the extent that the reception mechanisms that correspond to specific content properties are localized, one may not even need to resort to network-based analyses but could even rely on standard brain mapping-style analyses.

Then, as we move from simple features like brightness or sound energy to more complex media content, we need to not only adjust the kinds of content features that are quantified and used to model brain responses but also the kinds of brain response features that are modeled (i.e., moving from localized univariate response models to model networked responses and state-reorganizations, which is what network control theory excels in). With regard to the quantification of content, we argued above that it will no longer be sufficient to model pixels, brightness, or the occurrence of faces. Rather, media psychological research points to the importance of characters, the actions they perform and the outcomes that befall them, and so forth. Using this understanding (for a review, see Grizzard and Eden, 2022), the kinds of content we should attend to, and their putative brain targets become clearer. With regard to response features, we can rely on methods from network neuroscience, including parcellations of canonical brain networks, network estimation methods, and knowledge about structure–function relationships (e.g., between the TPJ, a core node of the DMN and social-affective processes, e.g., Yeshurun et al., 2017).

Imagine a researcher interested in empathy. Two narratives could be constructed, one where a liked character suffers a dramatic setback (which should elicit an empathetic response), and one where the setback is edited out (which should not elicit an empathetic response). The timing of this empathy-inducing outcome could be used in an impulse response model that targets a specific node in the network, like the temporal–parietal junction, which has long been implicated in empathy processing (Saxe and Kanwisher, 2003; Decety and Lamm, 2007; Alcalá-López et al., 2018).^11^ Then, one would analyze how this intervention (i.e., film event) changes the brain network topology and how this differs between the experimental and control version of the narrative. Moving onwards, if a negative event befalling a liked character changes the brain network into a state of empathy, then that change should be associated with a corresponding change in audience responses (e.g., self-reported empathy), thus completing the sequence from media content, reception response, to media effects.

Another example could be suspense: We know that suspense in media strongly affects the audience, and screenwriters and directors possess a lot of knowledge about how to elicit this phenomenon (e.g., Brewer and Lichtenstein, 1982; Douchet, 1985; Vorderer et al., 2006). Moreover, some prior work has focused on the brain mechanisms of suspense precisely because of its potential to take control of audiences (Bezdek et al., 2017; Schmälzle and Grall, 2020a,b). Much like in the example about empathy above, it would be possible to create different branches of the same story that incorporate directing techniques, music, narrative devices, or other methods to increase suspense and examine their impact on brain systems.^12^ Again, one could then analyze how variations in suspense (either between experimental conditions or variations of suspense over time) impact the brain network topology. One broad prediction, for example, is that ebbs and flows in suspense should impact the saliency and executive control networks, which are associated with attention. Although more difficult to resolve with present-day functional neuroimaging methods (because of limitations in spatial and temporal resolutions), suspense should also impact ascending arousal networks and cortico-subcortical loops associated with emotional arousal. Indeed, previous neuroimaging work points to such responses (e.g., Hermans et al., 2011; Naci et al., 2014; Young et al., 2017; Schmälzle and Grall, 2020a,b), but whereas much of this work is data-driven and more exploratory in nature, network control theory holds potential to integrate this research and provide a common platform for bringing together content (directors, creators), brain response (cognitive neuroscientists) and effects studies (media psychology and entertainment research).

These represent just a few possible examples that use network control theory as a framework that connects the domains of content analysis, reception analysis, and media effects. The appeal of network control theory is that it enables us to start from media-informed hypotheses about what will be driving brain network dynamics and how while honoring the complexity and hierarchical nature of the content (from simple objective features to more abstract semantic and pragmatic contents), brain responses (from evoked sensory responses to reorganization of higher-level brain systems), and media effects (from effects on individuals to populations, and from obligatory effects in all individuals to effects that could vary based on individual difference, cultural background, or an individual’s position in a larger social network topology).

Although many unknowns and challenges remain,^13^ this approach holds the potential to integrate domains that have henceforth been studied separately. Viewed from afar, this endeavor is almost reminiscent of the seminal work of Penfield (1950), who used intracranial stimulation techniques to map out functional brain systems, but with the difference that movies now offer a way to influence brain systems and associated affective, social, and conceptual reactions, and not only in individuals but multiple brains comprising an audience.^14^

## 4. Future directions

### 4.1. From traditional mass media to new media

We are not the first to make arguments about the necessity of quantifying naturalistic and multi-modal media stimuli for understanding the brain, or media effects (see, e.g., Weber et al., 2006, 2015b; Spiers and Maguire, 2007; Dudai, 2012; Sonkusare et al., 2019; Aliko et al., 2020; Finn et al., 2022). Important work headed in this direction already exists, and we have worked to note these developments at relevant points in our manuscript. The point is, however, this approach has not yet reached widespread adoption. We think this is for two key reasons: (1) uncertainty about how to quantify media content, and (2) ambiguity about how to link content’s complex, hierarchically organized, and time-varying effects across complex, hierarchically organized, and time-varying brain systems. The approach outlined above, which advocates jointly studying media content, reception responses, and media effects and suggests NCT as a framework for doing so, addresses these two challenges and is directly applicable to a wide variety of traditional mass media, including TV, cinema, and written or spoken narratives.

However, the notion of mass media today is no longer quite what it was when relevant definitions and theories of mass media were first formulated. Rather, these days the media ecosystem is constantly in flux, and new ways to stimulate brains and entertain audiences are constantly invented. Traditional mass media, most notably radio and television, followed the classical one-to-many model in which a sender emitted the same message that was carried *via* a medium to a large audience, like when people listened to Orson Wells’ “War of the Worlds” broadcast that prompted them to fear an alien invasion. Similarly, TV and cinema movie viewing also fall under this kind of paradigm (same message, millions of simultaneous receivers), which is very compatible with neuroimaging and leads to a constant increase in papers and publicly available datasets featuring audiobooks and movies (Aliko et al., 2020; Willems et al., 2020).

The advent of streaming platforms (e.g., Netflix for movies and shows, YouTube for all kinds of content, Spotify for music) prompted a shift in the landscape because previously more homogenous mass audiences became increasingly fragmented and can now consume content at their own pace and *via* increasingly niche content. Despite the self-timed nature of such video streaming, however, the basic notion of same-message - many receivers still remains. Thus, these kinds of media models lend themselves exceptionally well to neuroscientific studies like the ones outlined above.

Social media add another layer of complexity, but we argue that key principles of mass communication still remain relevant. Modern social media, like Twitter and TikTok, can be characterized as instant mass media; that is, they deliver the same messages to many recipients in a very swift manner. Moreover, they add novel affordances to engage with content *via* liking, sharing, and commenting. The resulting mode of communication has been called “masspersonal communication” because it blends elements of interpersonal communication into the mass communication model (O’Sullivan and Carr, 2018). Thus, the content of social media messages can still be studied and linked to brain reception responses, and the additional affordances of social media (like sharing, liking, commenting) can also be studied from a neuroscientific perspective (Meshi et al., 2015; Scholz et al., 2019).

### 4.2. Games and virtual reality as emerging trends

Reflecting on what the future may hold, we see two areas on the rise: Gaming and Virtual Reality (VR). Gaming and VR are both among the fastest-growing media types. Both offer interaction potential,^15^ distinguishing them from movies and stories (TV, radio, podcasts, etc.) that are consumed more passively, although even for the latter, audiences can vary in their level and degree of internal activity (e.g., interest, involvement, vigilance). At first glance, the interactive and thus constantly changing nature of gaming and VR media may seem incompatible with the “same stimulus sequence” notion that is so characteristic of movies, audiobooks, and other fixed-type mass media. However, we note that even in games and VR, there are clearly shared aspects as well and that the experiences users have are far from idiosyncratic. In games, for instance, many sub-scenes are prerecorded and thus the same for all audience members, and the same holds true for VR. Moreover, for both games and VR experiences, it is exceptionally well possible to quantify and precisely time-lock contents (Bente et al., 2007; Huskey et al., 2018a; Lammers et al., 2019; Calcagnotto et al., 2021).

Thus, although studying brain responses during games and VR will require special consideration, we argue again that the basic model outlined above still applies: As long as fixed content is consumed, one can code it just like one would do for movies or narratives (see above), and to the extent that content varies by person, one can still content-analyze each individual screen-recording using the same principles (Dmochowski et al., 2018; Huskey et al., 2018b, 2022; Ki et al., 2020).

## 5. Summary and conclusion

In sum, we have argued that the time is ripe for creating a new substantive science at the intersection of media and neuroscience. The neuroscientifically informed study of media reception processes provides the missing link between media content and media effects, enabling fascinating insights into the hidden mechanisms by which media affect us. However, to avoid reinventing the wheel or creating a mayfly-like field, neuroscientists should engage with research that has studied media content and media effects. The current article offers a springboard for doing so. We have introduced an organizing framework that connects the domains of media content, media reception, and media effects in a logical, sequential manner. In that framework, content is the key to understanding brain responses and subsequent media effects. We then suggested network control theory as a way to link the domains of media content, media reception mechanisms, and media effects (in individuals and social networks) in one multi-layered (or multi-staged) network. This framework offers a clear agenda for future research that uses media in combination with neural or other reception response measures and applies to studies focusing on specific neurocognitive processes (e.g., vision, language, or memory) as well as more integrative investigations of audience responses to movies and narratives. The ideas articulated here are most directly applicable to one-to-many mass communication models (which include neurocinematics, neuroscience of stories, etc.) but can also be adapted to modern social and interactive media.

## Author contributions

All authors listed have made a substantial, direct, and intellectual contribution to the work and approved it for publication.

## Conflict of interest

The authors declare that the research was conducted in the absence of any commercial or financial relationships that could be construed as a potential conflict of interest.

## Publisher’s note

All claims expressed in this article are solely those of the authors and do not necessarily represent those of their affiliated organizations, or those of the publisher, the editors and the reviewers. Any product that may be evaluated in this article, or claim that may be made by its manufacturer, is not guaranteed or endorsed by the publisher.

## Footnotes

^1^Of note, while we believe that upper levels (i.e., beyond sensory-perceptual content) are important and largely understudied, at least when it comes to neuroimaging, this does not mean that it was not worthwhile to study lower levels. For instance, researchers in visual neuroscience and neurolinguistics have both discovered the benefit of using movies as stimuli, and many other lower- and mid-level neurocognitive processes (e.g., event segmentation, situation model building, etc.) can be examined fruitfully using media.

^2^In fact, they may matter even more than lower-level content features because we can easily turn any story into a movie and vice versa, which completely exchanges the lower-level content types, but keeps the higher-level information intact (Honey et al., 2012; Regev et al., 2018). Likewise, one can also transform a given movie in many ways, like into a comic, or a reissue with newer actors, thus changing all lower-level features, yet it will still stay the same movie. However, we want to avoid creating the impression that we give priority to higher-level content elements or that we consider lower-level elements as less important. This is not the case. For example, content creators (directors and camera operators) often make strategic use of lower-level content elements (e.g., angle, shot sequence) to create specific impressions. Studying these techniques, their impact on brain activity, and their effects on viewers is as valuable as analyzing, e.g., the plot narrative.

^3^*Via* standard forward inference (Henson, 2006).

^4^Again, we are not shy to admit that the content properties that cater to these processes are difficult to quantify: For example, computing a sound envelope/RMSE feature is easy. Nowadays, computing BERT-embeddings for every word of a story is also quite doable. However, even though these properties are relevant to understanding a story, they alone are insufficient. Parallel arguments about this exact issue are also made in the NLP community, where debate rages about the capabilities and limitations of large-language models (Bender and Koller, 2020). Yet, again, we want to emphasize that our goal is not to declare only the plot level as the only level worth quantifying. Rather, examinations of specific linguistic and sound features, their creation, their effects on the brain, and their impact on audiences are inherently relevant and worth studying.

^5^Researchers often rely on their intuition. In fact, most movies used in fMRI studies seem to be chosen for their social-affective elicitation potential. Movies that have been used include Bang Bang! And you are dead; The Present; Partly Cloudy; Curb your Enthusiasm; The Office; Sherlock; Memento. These are all great, and it seems clear that "researchers felt something" when they opted to use these movies. However, none of the papers devoted more than one or two sentences to the content and theoretical reasons why it was chosen.

^6^Network science is an application of graph theory where systems of information can be grouped into nodes (specific elements) and edges (the relationships between those elements). The beauty of network science is its domain generality. Network systems can be constructed to represent social organization (e.g., each node is an individual, each edge represents if individuals are friends or not), information on the internet (e.g., nodes represent a webpage, edges represent hyperlinks between websites), civil infrastructure (e.g., nodes represent cities, edges represent highways connecting cities), biological systems (e.g., nodes represent gray matter corresponding to specific brain structures, edges represent white matter fiber tracts connecting gray matter), and more (for a review, see Newman, 2010). The constellation of edges connected by nodes describes a network’s organization. This organization is commonly referred to as a network’s topology.

^7^There is the energy necessary to maintain a given topological organization. In the case of brains and brain networks (both structural and functional), this is associated with energetic costs, most notably metabolic costs (Bullmore and Sporns, 2012). There is also the energy that is necessary to transition a system into, and maintain, a given topological organization. In neurscientific contexts, this includes things external energy sources such as an experimental task, a pharmacological intervention, a specific stimulus, and so on, that drive the brain from one functional topological organization to another. Network control theory can be used to account for both cases (see, e.g., Gu et al., 2015; Lydon-Staley et al., 2020, respectively). In our application, we are particularly focused on the latter case without denying the former.

^8^We thank a reviewer for pointing out that based on the picture presented here (content → brain → effects), readers may infer that media reception is a strictly passive process, which we ultimately do not believe to be correct. Rather, there are additional receiver-sided factors that can affect the reception process. For example, the degree of interest among receivers can modulate how people respond to the same incoming message content; the same is true for the belief-consistency of a message, the background knowledge audiences have about a topic, or simply their degree of vigilance. Thus, in reality, the way in which audiences select and engage with media content is going to be more dynamic, creating message-receiver interactions beyond simple message main effects, potential dynamic feedback loops, and other audience effects (e.g., during co-viewing vs. individual viewing). In sum, real audiences are more active (e.g., Biocca, 1988; Huskey et al., 2020) and these factors must be taken into account. Nevertheless, even if these (or other) additional external or internal factors come into play, it is clear that the proximal causal role of media content is critical and must be quantified.

^9^From the perspective of Moral Foundations Theory and the Model of Intuitive Morality and Media Exemplars, this could be considered a violation of the harm/care foundation (Tamborini, 2011; Graham et al., 2013).

^10^Readers who are familiar with traditional approaches to fMRI data analysis, such as the GLM framework, will realize that this approach is conceptually similar, the main difference being that it is applied here to network metrics as the dependent variable rather than to the activity of individual voxels.

^11^Of course, this approach requires a strong *a-priori* hypothesis. Data-driven approaches are also available. For instance, a researcher could, one by one, apply the energy source to each node in the network in a round-robin style, and observe the outcome.

^12^Of note, here we discuss only standard experimental paradigms, but it would not seem infeasible to even create closed-loop, neurofeedback-type systems that feedback audience activity into the creation process, thereby further enhancing collaboration and integration between filmmakers and neuroscience (e.g., Tikka et al., 2012; Raz and Hendler, 2014).

^13^Especially regarding the quantification of content that lies at or behind the "barrier of meaning" and the accurate measurement of the networked structures.

^14^We would like to thank a reviewer for suggesting that this all sounds a bit like a "content-powered TMS machine," an idea that we find thought-provoking and appropriate. However, the reviewer is also right to warn against overstretching this analogy because current TMS methods allow causal targeting of single (or few) and localized brain functions. By contrast, when content "targets" brain systems (such as the TPJ in the empathy example above), the TPJ would not receive direct input from content, but its input would consist of the preprocessed visual and auditory information conveying the empathy evoking narrative. Despite the caveats, we believe that the notion that media can precisely steer neurocognitive processes and evoke strong effects like emotional arousal, empathy, and so forth is convincing and that the NCT framework provides a way to examine how this is mediated by brain networks and their dynamic reconfigurations.

^15^Clearly, interactive media add immense complexity to the simple linear-causal content-reception-effects perspective offered for movies and comparable consumption media. Note that even for these types, however, there is interaction potential insofar as exposure to movies can shape preferences, thereby affecting future selection decisions and so forth. Thus, the overall picture is clearly more dynamic than described here. Yet, even a complex, convoluted episode with interactive media can be disentangled and causally arranged along the ‘arrow of time’, for which the content, reception, response framework should still hold.
